# Community-Acquired Pneumonia Caused by *Mycoplasma pneumoniae*: How Physical and Radiological Examination Contribute to Successful Diagnosis

**DOI:** 10.3389/fmed.2016.00028

**Published:** 2016-06-13

**Authors:** Tomoo Kishaba

**Affiliations:** ^1^Department of Respiratory Medicine, Okinawa Chubu Hospital, Uruma City, Okinawa, Japan

**Keywords:** mycoplasma, physical examination, auscultation, radiological findings, atypical

## Abstract

*Mycoplasma pneumoniae* is one of the most common causes of community-acquired pneumonia (CAP), particularly in young adults. Vital signs are usually normal except for temperature. On physical examination, general appearance is normal compared with that of typical pneumonia such as pneumococcal pneumonia patients. Mycoplasma sometimes causes ear infections such as otitis media. It is important to distinguish between typical pneumonia and atypical pneumonia such as mycoplasma pneumonia because having the right diagnosis allows for the use of the correct antibiotic to treat CAP while preventing development of drug-resistant bacteria and also decreasing medical cost. The symptoms and diagnosis of mycoplasma pneumonia is multi-fold. Auscultation of patients can demonstrate trace late inspiratory crackles or normal alveolar sounds; however, bilateral polyphonic wheezes can sometimes be heard because of bronchiolitis. With regard to radiological findings, a chest radiogragh often shows bilateral reticulonodular or patchy consolidation in both lower lobes. Pleural effusion is rarely observed in adult cases. Immunocompetent patients tend to reveal more extensive shadowing compared with immunocompromised patients. As serological diagnostic methods are not able to offer 100% reliable diagnosis, integration of physical and radiological examination is crucial to accurately diagnose mycoplasma pneumonia. Herein, I review the typical findings from physical examination and imaging patterns of patients with mycoplasma pneumonia.

## Introduction

*Mycoplasma pneumoniae* is one of the most common causes of community-acquired pneumonia (CAP), particularly in young adults (1, 2). Atypical agents including *M. pneumoniae* pneumonia account for 7–20% of CAP (3–7). There is a unique diagnostic criterion for atypical pneumonia in Japan. The Japanese Respiratory Society (JRS) propose six parameters such as young age, absence of underlying disease, an intractable or non-productive cough, absence of crackles, and absence of leukocytosis as criteria for atypical pneumonia, particularly mycoplasma pneumonia (8). According to a Japanese multi-center study including 403 typical pneumonia cases, 62, 46, and 13 cases were caused by *M. pneumoniae*, *Chlamydophila pneumoniae*, and *Chlamydophila psittasi*, respectively. The sensitivity and specificity of the JRS criteria were 88.6 and 69.8%, respectively, when only considering those aged <60 years (9).

A single high Mp-specific antibody titer is suggestive of recent infection; however, if the patient has been at hospital for less than a week, antibodies are usually negative. However, an increase in antibody titers by a factor of >4 in serum samples obtained during the acute and convalescent phases of the disease is indicative of a recent infection. In practice, this requires weeks of monitoring and is not practical. On the other hand, ImmunoCard (IC) Mycoplasma (Meridian) can detect IgM antibodies although this requires 3 or 4 days until a positive result is obtained. Furthermore, once it is positive, it will remain for some time. Ueda et al. reported that 31.3% IC Mycoplasma-positive cases showed a discrepancy when comparing the result of IC with that of the complement fixation (CF) test (10). Therefore, practical diagnosis requires a more rapid and reproducible test. Recently, more rapid detection of the Mycoplasma DNA, such as loop-mediated isothermal amplification (LAMP) system, has been reported that it is highly sensitive (11–14). Still, both physical findings and radiological patterns contribute to practical diagnosis of mycoplasma pneumonia. Herein, I review the typical physical findings and imaging patterns of mycoplasma pneumonia. In addition, I describe the crucially important differential diagnoses.

**HISTORY TAKING**: familial or school outbreaks of mycoplasma infection do occur, therefore, a detailed history regarding sick contacts are crucial for diagnosing mycoplasma infection.

## Vital Signs

Blood pressure and respiratory rate are usually within the normal range in mycoplasma infection, the patient will sometimes show pulse–temperature dissociation. However, relative bradycardia is observed less often compared with other atypical agents such as typhoid fever, Legionellosis, psittacosis, and rickettsia infection. Therefore, relative bradycardia has a low sensitivity in diagnosing mycoplasma pneumonia. The fever range is from a low to high grade such as 39°C (15–18).

## Physical Examination

### General

General appearance is the initial part of physical inspection. In patients with mycoplasma infection, the general appearance is normal compared with that in patients with typical pneumonia such as pneumococcal pneumonia and that caused by *Klebsiella pneumoniae* pneumonia.

### Ear

We sometimes observe ear pain or transient deafness because of otitis media caused by mycoplasma (19, 20), although the deafness is usually unilateral and reversible. Clinical symptoms of otitis media caused by mycoplasma include ear pain without discharge, which is different from exudative otitis media. In addition, severity of pain is mild rather than bacterial infection. Because the nose, sinuses, ear, pharynx, and lower airway are connected, once an organ is affected by mycoplasma infection, we sometimes observe that sinusitis or otitis media coexist with the pneumonia (21). Frontal headache and tenderness is associated with sinusitis. And mycoplasma patients sometimes notice sore throat as presenting symptom of Mycoplasma-related pharyngitis.

### Neck

Mycoplasma patients rarely show significant lymphadenopathy. Therefore, Epstein–Barr (EB) virus infection, acute human immunodeficiency virus (HIV), connective tissue disease (CTD), such as systemic lupus erythematosus (SLE), rheumatoid arthritis (RA), and adult-onset Still’s disease (AOSD) need to be ruled out. If mycoplasma infection involves the posterior pharynx or tympanic membrane, non-prominent cervical adenopathy can be observed. Therefore, lymphadenopathy of mycoplasma infection shows without tenderness and hardness.

### Lung

Majority of mycoplasma infection reveal only airway infection with intractable cough. However, only <10% showed pneumonia (22). Patients can exhibit either trace late inspiratory crackles or normal alveolar sound. Norisue et al. reported that among 74 mycoplasma patients, 58.1% showed no crackles and 33.8% showed late inspiratory crackles (23). Besides, the positive predictive value (PPV) of pneumococcal pneumonia (*n* = 43) and mycoplasma pneumonia (*n* = 74) based on crackles were 91 and 76%, respectively (Table 1). This means mycoplasma exclusively invades the airway or peribronchial interstitium without alveolar involvement. Therefore, most patients show an intractable, non-productive cough in the early phase. Sputum color change occurs late in the course. In addition, we sometimes detect bilateral polyphonic wheezes because of bronchiolitis (24). Presence of bronchiolitis is associated with hyperinflation or volume loss in radiological findings. Therefore, integration of auscultation and radiological findings provide useful information for pathogenesis of mycoplasma pneumonia. Mycoplasma patients experience dyspnea less often because the main target is the peribronchovascular interstitium and respiratory bronchiole, but not the alveolar septum. If patient report dyspnea, we consider pleural effusion or co-existence of asthma attack. Based on Cochrane Database Systematic Review, presence of chest pain is more than double the probability of Mp pneumonia. Wheeze was 12% more likely to be absent in children with Mp pneumonia [pooled positive likelihood ratio (LR+) 0.76, 95% CI 0.60–0.97; pooled negative likelihood ratio (LR−) 1.12, 95% CI 1.02–1.23] (25). Chest pain and wheeze are both useful symptoms contributing to diagnosis of Mp pneumonia both in children and in adolescents.

**Table 1:** **Sensitivity and specificity values based on crackles**.

	Sensitivity (%)	Specificity (%)	Positive predictive value (%)	Negative predictive value (%)
Pneumococcal pneumonia (*n* = 43)	83.1	85.7	90.7	75.0
Mycoplasma pneumonia (*n* = 74)	80.0	84.7	75.7	87.7

*Ref. (23)*.

### Heart

Rhythm is regular and no extra sounds are heard, although patients can sometimes exhibit a rhythm disturbance with palpitation (18). We rarely observe mycoplasma-associated myocarditis, which is not fatal. And myocarditis has been reported rare autopsy reports. There are several case reports of mycoplasma that are associated with thrombosis of the heart. The pathogenesis is the formation of anticardiolipin antibodies (26). This suggests immunological-mediated reaction. This unusual presentation is associated with chest pain. The incidence and severity of most forms of cardiac involvement increase with age.

### Abdomen

Gastrointestinal tract involvement is rarely observed, and symptoms are non-specific, although hepatitis and pancreatitis are possible because of cross-reacting antibodies to *M. pneumonia* (16). Compared to Legionellosis, mycoplasma infection rarely cause diarrhea.

### Extremities

Common skin manifestation is macro-papular rash with itching as nodular erythema. These skin lesions are often detected on both thighs. The most severe form of skin involvement is Stevens–Johnson syndrome (SJS) and toxic epidermal necrolysis (27), which are characterized by fever, rash, skin detachment, and mucositis (28). Medications such as sulfonamides, antiepileptics, non-steroidal anti-inflammatory drugs (NSAIDS), and allopurinol are associated with SJS. Therefore, a detailed drug history is required. Other manifestations such as arthralgia or muscle pain are rarely observed. And Raynaud phenomenon can be seen in *M. pneumoniae* infection secondary to cold agglutination formation (29) (Table 2).

**Table 2:** **Physical findings and clinical entity**.

	Physical findings	Clinical entity
Ear, nose, throat	Ear pain without discharge	Otitis media
	Frontal headache	Sinusitis
	Sore throat	Pharyngitis
Neck	Mild lymphadenopathy without tenderness	Lymphadenitis
Lung	Late inspiratory crackles	Pneumonia
	Polyphonic wheeze	Bronchiolitis
Heart	Palpitation	Rhythm disturbance
	Chest pain	Myocarditis, thrombosis
Skin	Maculopapular or vesicular rash	Stevens–Johnson syndrome
Central nervous system	Headache, consciousness disturbance	Encephalitis
	Tenderness of forehead	Meningitis
	Neck stiffness	Guillain–Barrë Syndrome
	Progressive muscle weakness	Acute transverse Myelitis
	Back pain, paralysis	Acute disseminated encephalomyelitis
	Behavior change hemiparesis

### Central Nervous System

We observed that 0.1% of all mycoplasma patients sometimes show central nervous system involvement (30). Fever, headache, and consciousness disturbance suggest encephalitis (31). Tenderness of forehead and neck stiffness is associated with meningitis. In addition, Guillain–Barrë syndrome may develop from mycoplasma infection if patients show progressive muscle weakness initiating in the lower extremities (32, 33), although this complication is quite rare. However, early recognition of neurological abnormality is important for prevention of neurological sequelae. In addition, acute transverse myelitis (ATM) and immunological infection, such as acute disseminated encephalomyelitis (ADEM), can result in some of the most severe complications associated with mycoplasma infection (34). Key symptoms of ATM are acute back pain and paralysis. ADEM usually show behavior change and hemiparesis with monophasic. The pathogenesis of CNS involvement of mycoplasma infection remains unknown. Direct infection or immune-mediated reactions are possible mechanisms (30).

When we examine a mycoplasma patient, we should exclude potential diagnoses based on key symptoms and characteristic physical findings and consider the clinical epidemiology.

## Radiological Findings

The chest radiograph shows bilateral reticulonodular or patchy consolidation across both lower lobes. In addition, mycoplasma tends to spread at the respiratory bronchiole resulting in alveolar collapse. Therefore, important findings from a chest radiograph are elevation of the diaphragm or downward shift of the minor fissure associated with volume loss. In children, volume loss is often associated with plastic bronchitis (35). A pleural effusion is rarely observed in adult cases. An increased risk of multilobar opacities was found among older or male patients with *M. pneumoniae* pneumonia (odds ratio, 1.065 and 3.279; 95% confidence interval, 1.041–1.089 and 1.812–5.934; *p* < 0.001 and *p* < 0.001, respectively). Patients with *M. pneumoniae* pneumonia showing multilobar opacities or consolidation had a significantly longer hospital length of stay (r = 0.111, r = 0.275; *p* < 0.033, *p* < 0.001, respectively) (36). Chest computed tomography (CT) without contrast material shows bronchovascular bundle (BVB) thickening and centrilobular nodules (37, 38) (Table 3). These two findings are consistent with the affinity of mycoplasma to airway cilia and the bronchioles. Other findings are consolidation, atelectasis, and ground glass opacity (GGO). In summary, imaging findings of mycoplasma are variable, although volume loss is often observed. For evaluation of volume loss, a serial chest radiograph is quite useful compared with chest CT, although if we use CT for detecting volume loss, displacement of the major fissure or minor fissure is key sign.

**Table 3:** **Imaging findings from atypical pneumonia**.

	BVB thickening	Centrilobular nodule	Volume loss	Consolidation	Pleural effusion
Legionella pneumonia	+	−	+	+++ (inhomogeneous)	+++
Mycoplasma pneumonia	++	+++	+	+	+ (child)
Chlamydophila pneumonia	−	+	+	++	−

*Ref. (39)*.

*BVB, bronchovascular bundle; GGO, ground glass opacity*.

## Differential Diagnosis

### Legionella Pneumonia

With regard to Legionnaires’ disease, the patient usually exhibits focal consolidation at the initial phase with these shadows bilaterally spread across the lung field later. One of the most common findings is peribronchovascular consolidation and bilateral pleural effusion (38). Approximately 70% of the patients show pleural effusion within 1 week (40).

### Chlamydophila Pneumonia

Typical findings of chlamydophila pneumonia are pan-lobular or non-segmental consolidation (39). These radiological findings are similar to pneumococcal pneumonia. In addition, these patients sometimes show volume loss associated with organizing pneumonia.

### Viral Pneumonia

Generally, primary viral pneumonia shows bilateral GGO (41). First, influenza A pneumonia often shows bilateral GGO and reticulation. However, both centrilobular nodule and bronchovascluar bundle thickening are usually absent. Second, human metapneumovirus (HMV) typically causes peribronchial thickening and linear shadowing. But, centrilobular nodule is rarely seen. In immunocompromised patients, we sometimes observe cytomegalo virus (CMV) pneumonia. In CMV pneumonia, bilateral GGO and a random distribution of small nodules is usually observed because the spread of infection of CMV is hematogenous. On the other hand, centrilobular nodule is often seen in Mp pneumonia. With these typical findings based on anatomical location, radiological distinction is possible from Mp pneumonia.

### Pneumocystis Pneumonia

We sometimes observe pneumocystis pneumonia (PCP) in immunocompromised patients, particularly patients who receive prednisolone without PCP prophylaxis such as a sulfa-containing drug. Typical imaging patterns of PCP are bilateral perihilar GGO and reticulation, with these shadows usually showing peripheral sparing. However, HIV-associated PCP often causes multiple cavities in the upper lung field.

In conclusion, comprehensive examination of patients with mycoplasma pneumonia is quite important because these patients often show variable extra-pulmonary manifestations. From a radiological perspective, understanding the favorite site of infiltration in mycoplasma pneumonia is crucial, based on the understanding of mycoplasma pathogenesis and lung anatomy.

Using five senses for the comprehensive understanding of mycoplasma pneumonia is the key point.

## Author Contributions

The author wrote general findings from physical and radiological examination of patients with *Mycoplasma pneumoniae* pneumonia.

## Conflict of Interest Statement

The author declares that the research was conducted in the absence of any commercial or financial relationships that could be construed as a potential conflict of interest.
